# Young children with a minor traumatic head injury: clinical observation or CT scan?

**DOI:** 10.1007/s00431-022-04514-8

**Published:** 2022-06-24

**Authors:** Nicky Niele, Frans B. Plötz, Ellen Tromp, Bart Boersma, Maarten Biezeveld, Matthijs Douma, Katja Heitink, Gavin ten Tusscher, Hans B. van Goudoever, Marlies A. van Houten

**Affiliations:** 1 Department of Pediatrics, Tergooi Hospital, Blaricum, The Netherlands; 2 Department of Pediatrics, Amsterdam UMC, University of Amsterdam, Emma Children’s Hospital, Meibergdreef 9, 1105AZ Amsterdam, The Netherlands; 3 Department of Epidemiology and Statistics, St Antonius Hospital, Nieuwegein, The Netherlands; 4 Department of Pediatrics, Noordwest Ziekenhuisgroep, Alkmaar, The Netherlands; 5 Department of Pediatrics, OLVG, Amsterdam, The Netherlands; 6 Department of Emergency Medicine, Dijklander Ziekenhuis, Hoorn, The Netherlands; 7 Department of Pediatrics, Flevo Ziekenhuis, Almere, The Netherlands; 8 Princess Maxima Center for Pediatric Oncology, Utrecht, The Netherlands; 9 Department of Pediatrics, Dijklander Ziekenhuis, Hoorn, The Netherlands; 10 Department of Pediatrics, Spaarne Gasthuis, Haarlem en Hoofddorp, The Netherlands

**Keywords:** Computed tomography scan, Guidelines, Observation, Pediatric minor traumatic head injuries

## Abstract

Currently, in young children with minor traumatic head injuries (MTHI) classified as intermediate risk (IR), PECARN recommends clinical observation over computer tomography (CT) scan depending on provider comfort, although both options being possible. In this study, we describe clinicians’ choice and which factors were associated with this decision. This was a planned sub-study of a prospective multicenter observational study that enrolled 1006 children younger than 18 years with MTHI who presented to six emergency departments in The Netherlands. Of those, 280 children classified as IR group fulfilling one or more minor criteria, leaving the clinician with the choice between clinical observation and a CT scan. In our cohort, 228/280 (81%) children were admitted for clinical observation, 15/280 (5.4%) received a CT scan, 6/280 (2.1%) received a CT scan and were admitted for observation, and 31/280 (11%) children were discharged from the emergency department without any intervention. Three objective factors were associated with a CT scan, namely age above 2 years, the presence of any loss of consciousness (LOC), and presentation on weekend days.

*Conclusion*: In children with MTHI in an IR group, clinicians prefer clinical observation above performing a CT scan. Older age, day of presentation, and any loss of consciousness are factors associated with a CT scan.

**Table:** 

**What is Known:** *• Clinical decision rules have been developed in the management of children of different risk groups with minor traumatic head injury (MTHI)*. *• According to the Dutch national, clinical decision rules in children under 6 years of age up to 50% of children classify as intermediate risk (IR) and clinicians may choose between clinical observation and computed tomography (CT)*.	
**What is New:** *• In this IR group, clinical observation is chosen in 81% children with MTHI*. *• In the subgroup where clinicians performed a CT scan, children were older and presented more frequently on a weekend day, and more frequently consciousness was lost*.

**What is Known:**

*• Clinical decision rules have been developed in the management of children of different risk groups with minor traumatic head injury (MTHI)*.

*• According to the Dutch national, clinical decision rules in children under 6 years of age up to 50% of children classify as intermediate risk (IR) and clinicians may choose between clinical observation and computed tomography (CT)*.

**What is New:**

*• In this IR group, clinical observation is chosen in 81% children with MTHI*.

*• In the subgroup where clinicians performed a CT scan, children were older and presented more frequently on a weekend day, and more frequently consciousness was lost*.

## Introduction

Clinical decision rules have been developed to guide clinicians in their management of children with minor traumatic head injury (MTHI) [1–4]. In young children with MTHI classified as high risk, a computed tomography (CT) scan is recommended to rule out significant intracranial pathology. Currently, in young children with MTHI classified as intermediate risk, the international PECARN guideline recommends clinical observation over CT scan, whereas the Dutch national guideline recommends either clinical observation or CT scan depending on clinicians choice [4]. Both clinical observation and performing a CT scan of the head have several advantages and disadvantages. A CT scan is fast and painless and as described earlier, it is the golden standard to detect intracranial abnormalities after MTHI. The risk of clinically important brain injury in children in the IR group is low in the presence of one isolated risk factor therefore argue against CT scan [5]. Moreover, there are several concerns such as radiation-related malignancies later in life, detection and clinical interpretation of non-specific findings, and the possible need for sedation in this age group. Clinical observation avoids the aforementioned side effects but is associated with higher health care costs [6]. Furthermore, there is no current consensus on the duration of observation and literature on the psychological and financial effect of separation of family during clinical observation is also lacking.

Although literature on clinician’s choice is scarce, some data suggest clinicians prefer clinical observation rather than performing a CT scan in this young patient population. This could be due to subjective factors such as clinical experience of the clinician or parental preferences [7, 8]. Objective factors remain indistinct, where factors such as age of the child and type of primary attending physician age are suggested [8, 9]. Other objective factors favoring a CT scan could hypothetically be several clinical parameters including timing of emergency department presentation. A better insight in these objective factors could provide more unambiguity for clinicians in emergency department management [7].

The aim of this study is twofold. First, we describe clinicians’ choice between clinical observation and CT scan in young children with MTHI in an IR group. Secondly, we describe which factors are associated with this decision. This knowledge may help us to create more insight and optimize our Dutch national guidelines.

## Methods

### Study design and patients

This was a planned sub-study of a prospective multicenter observational study that enrolled 1006 children younger than 18 years with MTHI who presented to six emergency departments in The Netherlands between 1 April 2015 and 31 December 2016 [10].

### Guidelines

**Fig. 1:**
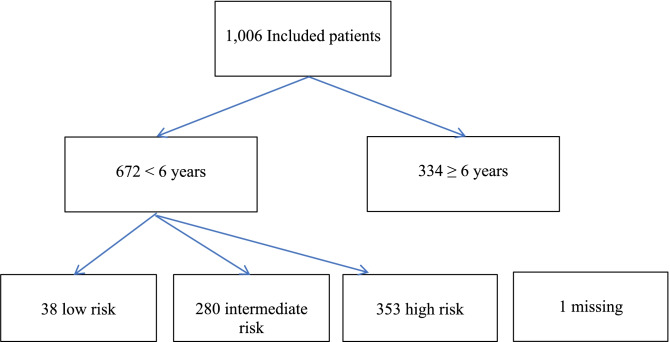
Patient’s enrollment

The Dutch national guidelines define several major and minor clinical criteria, specified by three age categories, namely under the age of two, between 2 and 5 years, and 6 years and older [4]. For children under the age of six in a high-risk group, a CT scan was recommended if they fulfilled one or more major criteria. If a child met one or more minor criteria, they were placed in an IR group and the clinician had the choice between a CT scan and clinical observation [4]. In children under the age of six, 353 children fulfilled one or more major criteria. Major criteria were CGS < 15, suspicion of a basilar skull fracture, posttraumatic seizures, focal neurological abnormalities, and high impact trauma of the head in all children under the age of six. In children under the age of two, scalp hematoma, a bulging anterior fontanelle, and ≥ five episodes of vomiting or vomiting more than 6 h after trauma were added as major criterion. In children aged 2–5 years, altered behavior and vomiting were classified as major criterion [4]. Minor criteria were loss of consciousness and a fall ≥ 1 m or ≤ 1 m with severe trauma mechanism in all children under six. In the youngest children under 2 years of age, a fall on hard ground, < five episodes of vomiting, altered behavior, and unknown trauma were additional minor criteria. In children aged 2–5 years old, presence of a headache was an additional minor criterion (Fig. 1).

### Data analysis

For the primary outcome, a comparison was made between the number of CT scans and the number of clinical observations in children under the age of six. The comparison was performed for the IR group, in which the choice was between a CT scan and clinical observation. For those 280 selected patients, the following data were extracted to evaluate which objective factors are associated with this choice: age, sex, Glasgow Coma Scale (GSC) at presentation, presence of any loss of consciousness, abnormalities at physical examination, time of day at injury, time of day at emergency department presentation, weekday or weekend day, primary responsible specialism (pediatrician, neurologist, surgeon, emergency physician, or others), and trauma mechanism.

### Statistical analyses

All statistical analyses were performed using SPSS version 22.0 (IBM Corp., New York, NY, USA). We reported descriptive statistics of all groups. The Kruskal–Wallis test was conducted to examine the differences between two or more groups (in actual emergency department management: CT scan, observation, CT scan and observation, or discharge). The Mann–Whitney *U* test was conducted for pairwise comparisons.

Missing data not excluded from analysis.

### Ethical statement

The study protocol for the original database study was approved by the Medical Ethics Review Committee North Holland (reference number NH014.229, registration number M014-040).

## Results

### Patient population

In our original cohort, 1006 children younger than 18 years with MTHI were enrolled of whom 672 children were younger than 6 years of age. Of those 672 children, 280 (42%) were classified as IR group fulfilling one or more minor criteria, leaving the clinician with the choice between clinical observation and a CT scan.

### Clinical observation vs. CT scan

In our cohort, 228/280 (81%) children were admitted for clinical observation, 15/280 (5.4%) received a CT scan, 6/280 (2.1%) received a CT scan and were admitted for observation, and 31/280 (11%) children were discharged from the emergency department without any intervention. All children who were admitted were discharged in good clinical condition. All CT scans showed no signs of traumatic brain injuries. None of the children returned after discharged from the ward or emergency department.

### Objective factors

In our cohort, the majority of children presenting in the emergency department were admitted for clinical observation. We therefore looked at factors which were associated with a CT scan. We had some missing data (21/280 no data on time of injury; 1/280 no data on loss of consciousness). We found three factors (Table 1). First, relatively more isolated CT scans were performed in the older age group (10/59, 17% in children aged 3–5 years) in comparison with the younger age group (4/37, 11% in children aged 2–3 years; 1/83, 1.2% in children aged 1–2 years; none of children < 1 year received a CT scan) (*p* < 0.001). Second, the day of presentation during weekend days children more often received a CT scan (7/189, 3.7% weekdays; versus 8/91, 8.8% weekend days). Third, a CT scan was more often performed in the presence of any LOC, namely in 5 of the 31 (16%), versus 10/248 (4.0%) in the absence of any LOC.

**Table 1:** Management of children under 6 years of age with MTHI classified as intermediate risk (IR) (*N* = 280)

Children < 6 years of age in an IR group with choice between observation and CT scan (*N* = 280)						
	Total *N* = 280 (%)	Isolated CT scan *N* = 15 (%)	Observation *N* = 228 (%)	CT and observation *N* = 6 (%)	Discharge *N* = 31 (%)	Significance
**Sex**						
Male	157 (56.1)	11 (7.0)	129 (82.2)	3 (1.9)	14 (8.9)	*p* = 0.332
Female	123 (43.9)	4 (3.3)	99 (80.5)	3 (2.4)	17 (13.8)	
**Age**						
< 1 y	101 (36.1)	0 (0)	93 (92.1)	2 (2.0)	6 (5.9)	*p* < 0.001
1–2 y	83 (29.6)	1 (1.2)	67 (80.7)	3 (3.6)	12 (14.5)	
2–3 y	37 (13.2)	4 (10.8)	27 (73.0)	0 (0)	6 (16.2)	
3–4 y	25 (8.9)	5 (20.0)	18 (72.0)	0 (0)	2 (8.0)	
4–5 y^§^	34 (12.1)	5 (14.7)	23 (67.6)	1 (2.9)	5 (14.7)	
**Primary responsible specialism**						
Pediatrician	146 (52.1)	12 (8.2)	113 (77.4)	3 (2.1)	18 (12.3)	*p* = 0.632
Neurologist	22 (7.9)	0 (0)	21 (95.5)	0 (0)	1 (4.5)	
Surgeon	48 (17.1)	1 (2.1)	41 (85.4)	2 (4.2)	4 (8.3)	
Emergency physician	63 (22.5)	2 (3.2)	52 (82.5)	1 (1.6)	8 (12.7)	
Other	1 (0.4)	0 (0)	1	0 (0)	0 (0)	
**Day of presentation**						
Weekday	189 (67.5)	7 (3.7)	162 (85.7)	5 (2.6)	15 (7.9)	*p* = 0.018
Weekend day^#^	91 (32.5)	8 (8.8)	66 (72.5)	1 (1.1)	16 (17.6)	
Time of injury*						
08.00–17.00	152 (58.7)	12 (7.9)	119 (78.3)	4 (2.6)	17 (11.2)	*p* = 0.352
17.00–08.00	107 (41.3)	3 (2.8)	88 (82.2)	2 (1.9)	14 (13.1)	
**Time of presentation**						
08.00–17.00	132 (47.3)	9 (6.8)	105 (79.5)	4 (3.0)	14 (10.6)	*p* = 0.568
17.00–08.00	147 (52.7)	6 (4.1)	122 (83.0)	2 (1.4)	17 (11.6)	
**Trauma mechanism**						
Fall ≤ 1 m	130 (47.1)	6 (4.6)	104 (80.0)	1 (0.8)	19 (14.6)	*p* = 0.227
Fall > 1 m	146 (52.9)	9 (6.2)	121 (82.9)	4 (2.7)	12 (8.2)	
Pedestrian/bicyclist vs. motor vehicle	3 (1.1)	0 (0)	2 (66.7)	0 (0)	1 (33.3)	*p* = 0.646
No pedestrian/bicyclist vs. motor vehicle	277 (98.9)	15 (5.4)	226 (81.6)	6 (2.2)	30 (10.8)	
Discharge from motor vehicle	0					1
No discharge from motor vehicle	280 (100)	15 (5.4)	228 (81.4)	6 (2.1)	31 (11.1)	
Other	29 (10.4)	3 (10.3)	20 (69.0)	0 (0)	6 (20.7)	*p* = 0.139
No other	251 (89.6)	12 (4.8)	208 (82.9)	6 (2.4)	25 (10.0)	
Any loss of consciousness*						
Yes	31 (11.1)	5 (16.1)	24 (77.4)	1 (3.2)	1 (3.2)	*p* = 0.022
No	248 (88.9)	10 (4.0)	203 (81.9)	5 (2.0)	30 (12.1)	

^§^Children aged both 4 and 5 years old

^#^Defined as Saturday and Sunday and all Dutch feast days

^*^Some missing data

All other objective data showed no statistical difference between the decision for a CT scan or clinical observation.

## Discussion

This is the first study to report clinical observation and CT rates in children under 6 years of age with MTHI in an IR group, favoring clinical observation in this young age group. In addition, we show three objective factors associated with a CT scan, namely age above 2 years, the presence of any LOC, and presentation on weekend days.

The preference for observation in the very young age group could be due to historical concerns of radiation exposure in this group. Young infants are more susceptible to radiation-related malignancies than adults and have a longer lifespan to express late effects. Overall average medical radiation effective dose has increased up to sevenfold over the last decades [11]. Therefore, children should be scanned with the lowest dosage as possible if a head CT scan is warranted. Present CT imaging protocols of the head in children deliver 0.6–2.0 mSv [12] in effective radiation dose to the head. This is lower than the current range of background radiation exposure, raising the question if this is still clinically relevant. In total, 120 new pediatric brain tumors are diagnosed each year in The Netherlands [13]. A nationwide study performed in 168,000 pediatric patients, who received one or more head CT scans between 1979 and 2012, found that the decade after the first head CT scan, one excess case per 10,000 head CT scans is estimated to occur [14]. Annually, more than 12,000 children are seen in Dutch emergency departments with MTHI [4] of which 3300 estimated (280/1006 × 12,000) are under the age of 6 and classify as IR. If clinicians would prefer a CT scan in all of these children, this would lead to one brain tumor every 3 years, a notable contribution to the total amount of pediatric brain tumors.

Clinical observation does not have this risk, but uniform execution is difficult since there is no consensus on the duration of clinical observation. Historically the length of stay varied between 12 and 48 h after trauma. In 1999, the American Academy of Pediatrics recommended that duration of observation would extend at least 24 h to look for signs of neurologic deterioration, but could be accomplished in any location (ED, hospital, or at home) [15]. Deterioration is typically due to increased intracranial pressure from either an expanding intracranial hematoma or cerebral edema. Epidural hematomas are rare, but associated with a lucid interval after which symptoms develop [16]. In a retrospective cohort of more than 17,500 children with MTHI, no child had a diagnosis of intracranial hemorrhage more than 6 h after trauma, suggesting that the vast majority will become apparent in this period [17]. Regarding the pathophysiology and aforementioned literature strategies of some clinics to discharge children 6 h after trauma in disconcordance with our national guideline can be defended.

Secondly, we showed three objective factors associated with a CT scan, namely age above 2 years, the presence of any LOC, and presentation on weekend days. Until now, there has been no literature on objective factors and emergency department management in young children in an IR group with MTHI. Overall, it has been previously described that type of primary attending physician and race or ethnicity are associated with performing head CT scans [8, 18]. Performing more CT scans in the older children could be due to practical feasibility which is difficult in the young child together with radiation exposure risk. Secondly, the preference for CT scanning in the presence of any LOC seems obvious. Nevertheless, all pediatric guidelines for MTHI incorporate this risk factor differently. Our Dutch guidelines and the PECARN rule define LOC as an IR, and thereby leave the choice between observation and performing a CT scan to the clinician [1, 4]. The CHALICE guideline adds a time factor, advising to perform a CT scan if the witnessed LOC exists longer than 5 min [2]. The Canadian guidelines do not define LOC as a risk factor, but as part of the definition for MTHI [3]. This raises the question whether isolated LOC is a solitary risk factor for intracranial traumatic brain injury. Literature states that this is a high-risk factor in adults, but not in children. Hereby, classifying it as an IR risk factor is a safe choice, but it is understandable that clinicians prefer a CT scan over observation [19, 20]. The final described objective factor associated with a CT scan is presentation of the young child on weekend days. In the literature, this last phenomenon is called the “weekend” effect. In adults with traumatic head injury, a higher mortality rate has been described during the weekends. However, mechanisms up until now behind this effect must still be determined [21]. Evidence for this phenomenon in children with MTHI is lacking. One could hypothesize that CT scanning is the first choice or more often chosen due to the current hospitalization capacity especially during the weekends or the absence or presence of certain specialties during weekend hours.

Although we highlighted three objective factors associated with a CT scan, we did not examine the rational for this preference, therefore making it difficult to incorporate them in current guidelines. Yet, it would be preferable to provide clinicians better tools to make a solid choice between observation and CT scan in this large group of children. Namely up to 50% of young children under 6 years of age with MTHI classify as IR [22]. Literature shows there are several subjective factors guiding the decision of clinicians, for example clinical experience of the clinician [7] and parental preference [8]. Natale et al. showed that regardless of the risk for clinically important traumatic head injury, parental anxiety and request was a common factor influencing clinician’s decision, especially in children of white non-Hispanic race/ethnicity [18]. This has also been shown by Ishida et al. In nearly 40% of children in a low-risk group for intracranial abnormalities, a CT scan was performed if parents “favored” one. This is in contrast to only 2% of children in this risk group if the decision was deferred to the clinician [8]. In addition to reduce CT scans, Hess and colleagues showed that shared decision-making led to more knowledge, less decisional conflict for parents, and better involvement than usual care [23]. It also endorsed greater trust in their clinicians. We would recommend incorporating shared decision-making in our current guidelines to incorporate family preferences into decision-making algorithms when the course of action for children with MTHI is unequivocal.

Our study has several limitations. It is an analysis of a relatively small group of children with overall a low percentage of CT rates. In a bigger cohort, it would have been possible to extract more objective factors associated with a CT scan. Secondly, we did not ask clinicians their argument for their choice. Therefore, we can only describe objective factors that suggest a CT scan in an IR group of young children with MTHI, which could potentially influence clinicians’ choice. However, specific individual reasons remain unknown.

## Conclusion

Our study demonstrates that clinicians prefer clinical observation above performing a CT scan in young children with MTHI in an IR group. In addition, we found three factors which are associated with this decision. However, since there is no rational for these factors, caution is advised to incorporate them in current guidelines.

## Data Availability

Not applicable.

## Abbreviations

CT: Computed tomography
IR: Intermediate risk
LOC: Loss of consciousness
MTHI: Minor traumatic head injuries

## References

[1] Kuppermann N, Holmes JF, Dayan PS (2009). Identification of children at very low risk of clinically-important brain injuries after head trauma: a prospective cohort study. Lancet.

[2] Dunning J, Daly JP, Lomas J-P (2006). Derivation of the children’s head injury algorithm for the prediction of important clinical events decision rule for head injury in children. Arch Dis Child.

[3] Osmond MH, Klassen TP, Wells GA (2010). CATCH: a clinical decision rule for the use of computed tomography in children with minor head injury. CMAJ.

[4] Hageman G, Pols MA, Schipper DM et al (2010) Richtlijn Opvang Van Patiënten Met Licht Traumatisch Hoofd / Hersenletsel. Utrecht: Nederlanse Vereniging Neurologie. https://richtlijnendatabase.nl/richtlijn/licht_traumatisch_hoofd_hersenletsel_lth/licht_traumatisch_hoofd_hersenletsel_-_startpagina.html

[5] Nigrovic LE, Kuppermann N (2019). Children with minor blunt head trauma presenting to the emergency department. Pediatrics.

[6] Nishijima DK, Yang Z, Urbich M (2015). Cost-effectiveness of the PECARN rules in children with minor head trauma. Ann Emerg Med.

[7] Niele N, Willemars L, van Houten M, Plötz FB (2018). National survey on managing minor childhood traumatic head injuries in the Netherlands shows low guideline adherence and large interhospital variations. Acta Paediatr.

[8] Ishida Y, Manabe A, Oizumi A (2013). Association between parental preference and head computed tomography in children with minor blunt head trauma. JAMA Pediatr.

[9] Niele N, Willemars L, van Houten M, Plötz FB (2019) Large variety of medical specialties involved in management of pediatric minor traumatic head injury in the Netherlands. Glob Pediatr Heal 6:2333794X1984611. 10.1177/2333794x19846117 10.1177/2333794X19846117 PMC6487757 31065577

[10] Niele N, van Houten MA, Boersma B (2019). Multi-centre study found that strict adherence to guidelines led to computed tomography scans being overused in children with minor head injuries. Acta Paediatr.

[11] National Council on Radiation Protection and Measurements (2009) Ionizing radiation exposure of the population of the United States. Bethesda, MD: NCRP; Report No. 160. https://ncrponline.org/publications/reports/ncrp-report-160-2

[12] Sheppard JP, Nguyen T, Alkhalid Y et al (2018) Risk of brain tumor induction from pediatric head CT procedures: a systematic literature review. Brain Tumor Res Treat 6:1. 10.14791/btrt.2018.6.e4 10.14791/btrt.2018.6.e4 PMC5932294 29717567

[13] Reedijk AMJ, van der Heiden-van der Loo M, Visser O  (2017). Site of childhood cancer care in the Netherlands. Eur J Cancer.

[14] Meulepas JM, Ronckers CM, Smets AMJB (2019). Radiation exposure from pediatric CT scans and subsequent cancer risk in the Netherlands. J Natl Cancer Inst.

[15] The management of minor closed head injury in children (1999) Committee on quality improvement, American Academy of Pediatrics. Commission on Clinical Policies and Research, American Academy of Family Physicians. Pediatrics 104(6):1407–15. PMID: 10585999 10585999

[16] Schutzman SA, Barnes PD, Mantello M, Scott RM (1993). Epidural hematomas in children. Ann Emerg Med.

[17] Hamilton M, Mrazik M, Johnson DW (2010). Incidence of delayed intracranial hemorrhage in children after uncomplicated minor head injuries. Pediatrics.

[18] Natale JAE, Joseph JG, Rogers AJ (2012). Cranial computed tomography use among children with minor blunt head trauma: association with race/ethnicity. Arch Pediatr Adolesc Med.

[19] Foks KA, Dijkland SA, Lingsma HF (2019). Risk of intracranial complications in minor head injury: the role of loss of consciousness and post-traumatic amnesia in a multi-center observational study. J Neurotrauma.

[20] Lee LK, Monroe D, Bachman MC (2014). Isolated loss of consciousness in children with minor blunt head trauma. JAMA Pediatr.

[21] Schneider EB, Hirani SA, Hambridge HL (2012). Beating the weekend trend: increased mortality in older adult traumatic brain injury (TBI) patients admitted on weekends. J Surg Res.

[22] Niele N, van Houten M, Tromp E (2020). Application of PECARN rules would significantly decrease CT rates in a Dutch cohort of children with minor traumatic head injuries. Eur J Pediatr.

[23] Hess EP, Homme JL, Kharbanda AB (2018). Effect of the head computed tomography choice decision aid in parents of children with minor head trauma: a cluster randomized trial. JAMA Netw open.

